# Breakthrough Rectal *Neisseria gonorrhoeae* Infections After Meningococcal B Vaccination: Microbiological and Clinical Features

**DOI:** 10.1093/ofid/ofae562

**Published:** 2024-11-04

**Authors:** Angelo Roberto Raccagni, Sara Diotallevi, Riccardo Lolatto, Elena Bruzzesi, Maria del Carmen Garcia Martearena, Ilaria Mainardi, Caterina Candela, Diana Canetti, Girolamo Piromalli, Nicola Clementi, Roberto Burioni, Antonella Castagna, Silvia Nozza

**Affiliations:** Infectious Diseases Unit, Vita-Salute San Raffaele University, Milan, Italy; Infectious Diseases Unit, IRCCS San Raffaele Scientific Institute, Milan, Italy; Infectious Diseases Unit, IRCCS San Raffaele Scientific Institute, Milan, Italy; Infectious Diseases Unit, IRCCS San Raffaele Scientific Institute, Milan, Italy; Infectious Diseases Unit, Vita-Salute San Raffaele University, Milan, Italy; Infectious Diseases Unit, Vita-Salute San Raffaele University, Milan, Italy; Infectious Diseases Unit, Vita-Salute San Raffaele University, Milan, Italy; Infectious Diseases Unit, Vita-Salute San Raffaele University, Milan, Italy; Infectious Diseases Unit, IRCCS San Raffaele Scientific Institute, Milan, Italy; Infectious Diseases Unit, IRCCS San Raffaele Scientific Institute, Milan, Italy; Infectious Diseases Unit, Vita-Salute San Raffaele University, Milan, Italy; Laboratory of Microbiology and Virology, IRCCS San Raffaele Scientific Institute, Milan, Italy; Infectious Diseases Unit, Vita-Salute San Raffaele University, Milan, Italy; Laboratory of Microbiology and Virology, IRCCS San Raffaele Scientific Institute, Milan, Italy; Infectious Diseases Unit, Vita-Salute San Raffaele University, Milan, Italy; Infectious Diseases Unit, IRCCS San Raffaele Scientific Institute, Milan, Italy; Infectious Diseases Unit, Vita-Salute San Raffaele University, Milan, Italy; Infectious Diseases Unit, IRCCS San Raffaele Scientific Institute, Milan, Italy

**Keywords:** 4CMenB, gonorrhoea, meningococcus, *Neisseria gonorrhoeae*, vaccine

## Abstract

**Background:**

4CMenB appears to be effective in reducing *Neisseria gonorrhoeae* (Ng) infections. Aims are to assess factors associated with breakthrough rectal Ng after 4CMenB and evaluate clinical and microbiological characteristics of breakthrough infections compared with before vaccination.

**Methods:**

This was a retrospective study of gay, bisexual, and other men who have sex with men (GBMSM) vaccinated with 4CMenB (2 doses) between 2017 and 2023 at the San Raffaele Scientific Institute for Research, Hospitalization and Healthcare (IRCCS San Raffaele Scientific Institute), Milan, Italy, and tested for rectal Ng. Rectal Ng infection is considered breakthrough if it occurs >1 month after the second 4CMenB dose and with positive nucleic acid amplification test (NAAT) result. Follow-up was from July 2017 (first 4CMenB vaccination) to November 2023 (data freeze). Rectal Ng was screened with both NAAT and gonococcal-specific cultures. Characteristics of individuals with or without breakthrough Ng and of Ng infections before or after 4CMenB were compared using Mann-Whitney and χ^2^/Fisher tests.

**Results:**

Overall, 473 GBMSM vaccinated with 4CMenB were included, with a median age (interquartile range) of 43 (37–51) years; 451 of 473 were living with human immunodeficiency virus. The percentage of NAAT-positive rectal Ng swab samples was 76 of 957 (7.7%) after 4CMenB and 51 of 456 (11.1%) before. Breakthrough rectal Ng after baseline were 76 in 57 of 473 people. People with rectal Ng after 4CMenB were younger, more likely to have a previous sexually transmitted infection, and had more sexual partners than those without (all *P* < .001). Breakthrough rectal Ng infections were less frequently symptomatic (34.2% vs 66.7%; *P* = .001) and more likely with negative gonococcal-specific culture (55.3% vs 19.6%; *P* < .001) compared with before vaccination.

**Conclusions:**

Breakthrough rectal Ng infections after 4CMenB were 76 in 57/473 people, preferentially identified in GBMSM with higher-risk sexual behaviors, were less often symptomatic, and more often with negative gonococcal-specific cultures, suggesting lower infection virulence.

## Teaser

Breakthrough rectal *Neisseria gonorrhoeae* infections following 4CMenB vaccination among gay, bisexual, and other men who have sex with men were preferentially identified in people with higher-risk sexual behaviors, were less often symptomatic and more often associated with negative gonococcal-specific culture.

*Neisseria gonorrhoeae* (Ng) is a globally prevalent sexually transmitted infection (STI) that poses significant challenges due to increasing antimicrobial resistance and rising numbers of cases [1]. To date, no effective vaccine is available that directly targets Ng, although the safety and efficacy of the investigational Ng generalized modules for membrane antigens vaccine is currently being evaluated [2]. In this scenario, the already licensed multicomponent meningococcal serogroup B vaccine (4CMenB) may be effective in reducing the risk of Ng infection [3, 4]. Promising cross-protection against gonorrhoea has been documented in several retrospective evaluations of its effectiveness, particularly among members of key populations such as gay, bisexual, and other men who have sex with men (GBMSM) [5–9]. For example, people with human immunodeficiency virus (HIV) and HIV preexposure prophylaxis (PrEP) users are key populations disproportionately affected by Ng [4].

Early evidence from New Zealand suggests that the outer membrane vesicle–based serogroup B *Neisseria meningitidis* vaccine MeNZB could provide an expected 31% efficacy against Ng [10, 11]. The currently used 4CMenB vaccine contains the same outer membrane vesicles as MeNZB, along with another antigen, *Neisseria* heparin-binding antigen, which is also present on gonococcal surfaces and may provide even greater protection against Ng [3]. Retrospective studies in Canada, Australia, and the United States have shown a lower incidence of gonorrhoea in people vaccinated with 4CMenB, with estimated efficacy ranging from 33% to 59% [5–8]. In addition, cost-effectiveness analyses also support these potential vaccination strategies, focusing primarily on higher-risk populations such as GBMSM to reduce Ng cases, with potential benefits at the community level [12]. Finally, the effectiveness of vaccination is also supported by both biological rationale and evidence of in vitro and in vivo detection of cross-reactive antibodies [13, 14]. In light of this evidence, the Joint Committee on Vaccination and Immunisation, the UK government's scientific advisory committee on vaccination policy, has recommended the off-label use of 4CMenB to reduce gonorrhoea cases among members of key populations at increased risk of Ng [3, 15].

In this context, White et al [16] published a commentary on the importance of evaluating the estimated gonorrhoea vaccine protection and symptomaticity according to anatomic site, given the lack of available data, in order to correctly inform vaccination policies. At our center, the Infectious Diseases Unit of the San Raffaele Scientific Institute for Research, Hospitalization and Healthcare (IRCCS San Raffaele Scientific Institute), Milan, Italy, our group previously conducted a retrospective case-control study to assess the effectiveness of 4CMenB vaccination against Ng in the specific key population of GBMSM with HIV [9]. Overall, we found a vaccine effectiveness of 42% against Ng after 2 doses of 4CMenB, in line with other observational studies previously mentioned [9]. Given the importance of obtaining further data, as highlighted by White et al [16], we undertook an observational study focusing on rectal Ng, given the high incidence of infection at this site in GBMSM. The aims of this study are to assess factors associated with the presence of breakthrough rectal Ng infections following 4CMenB vaccination and to evaluate the clinical and microbiological characteristics of breakthrough rectal Ng infections compared with those before 4CMenB vaccination in GBMSM.

## METHODS

This is a retrospective study of GBMSM aged ≥18 years who reported receptive sex and were receiving HIV care or a prescription for HIV PrEP at the Infectious Diseases Unit of the IRCCS San Raffaele Scientific Institute in Milan. People vaccinated with 2 doses of 4CMenB (according to international recommendations, with the timing of the second 4CMenB dose between 1 and 6 months after the first) between July 2017 (date of first 4CMenB vaccination) and November 2023 (data freeze) were included. 4CMenB vaccination was offered to people with HIV based on Italian HIV care guidelines due to increased risk of meningococcal disease; more recently, PrEP users were also offered 4CMenB vaccination after medical counselling on the expected benefits, based on the increased risk of meningococcal disease among GBMSM and the existing evidence on the effectiveness of 4CMenB vaccination against Ng [3, 17]. All vaccinations were administered directly by the referring physician at the Infectious Diseases Unit of IRCCS San Raffaele Scientific Institute in Milan.

In accordance with international guidelines, people were tested for rectal Ng if they reported specific symptoms suggestive of Ng infection or during routine asymptomatic screening (ie, every 3 or 6 months) as part of HIV and PrEP clinical care. All individuals fully vaccinated with 4CMenB with available follow-up and ≥1 screening for Ng at the rectal site were included in the study. Ng testing was performed at the Laboratory of Microbiology and Virology, IRCCS San Raffaele Scientific Institute, Milan, using concurrently Cobas 6800 Roche CT/NG nucleic acid amplification testing (NAAT) and gonococcal-specific cultures on rectal swabs (the latter as part of antimicrobial resistance surveillance). Specifically, for Ng cultures, all strains were plated on GC agar and CG agar supplemented with 1% hemoglobin and 1% IsoVitaleX enrichment (43611; Biomerieux). Plates were then incubated at 37°C with 5% carbon dioxide for 20–24 hours. A Clinical and Laboratory Standards Institute–recommended quality control strain was obtained from the American Type Culture Collection (ATCC 49226) and propagated according to ATCC guidelines. Individuals with multiple Ng infections at different time points were considered and counted as separate episodes.

Rectal Ng infection was considered breakthrough if it occurred >1 month after the second dose of 4CMenB (baseline) and with a positive NAAT, based on previous similar studies [5, 6]. Positive gonococcal culture was not considered necessary to demonstrate Ng infection (ie, microbiologically confirmed was defined as positive NAAT with either positive or negative gonococcal culture). Symptomatic rectal Ng infection was defined as the presence of one of the following: discharge, rectal bleeding, tenesmus, pain, constipation, diarrhea, or pruritus.

Individual characteristics (ie, clinical and vaccination history, previous STIs, sexual behavior, microbiological and laboratory data) were recorded during routine clinical care visits in the database of the Infectious Diseases Unit of the IRCCS San Raffaele Scientific Institute, Milan (Centro San Luigi Cohort). This cohort was approved by the Ethics Committee of San Raffaele Hospital (4 December 2017; protocol 34). At their first visit, individuals provide written informed consent for the use of their anonymized data in scientific analyses. Individuals included in the study signed written informed consent to be included in the published version of the manuscript in all formats and to be included in the Centro San Luigi Cohort.

The study was conducted and reported in accordance with the Declaration of Helsinki. Recorded data were anonymized and managed in accordance with good clinical practice.

Information on previous and concurrent microbiologically confirmed STIs, together with the respective infection site, were retrieved from our database, including *Chlamydia trachomatis* (Ct), syphilis, genital herpes simplex virus (HSV) 1/2, mpox, and *Mycoplasma genitalium* (Mgen) [Mgen was tested for only if symptoms were present]. Information on the sexual behavior of included individuals, such as the number of sexual partners in the 3 months prior to baseline and the type of sexual intercourse (ie, condomless receptive intercourse as study inclusion criterion), was self-reported to the referring physician.

Descriptive analyses of the individual characteristics were performed, using median (interquartile range [IQR]) and frequency (percentage). Characteristics of individuals with or without breakthrough rectal Ng and the characteristics of rectal Ng infections before and after 4CMenB vaccination were compared using χ^2^/Fisher exact test or the Mann-Whitney test for categorical and continuous variables, respectively. As some studies have suggested that chlamydia coinfection may reduce the effectiveness of 4CMenB against Ng infection, we compared the characteristics of rectal Ng infections after 4CMenB between people with or without concurrent rectal Ct at the time of breakthrough rectal Ng, using the χ^2^ test [10]. Incidence rates (IRs) and corresponding 95% confidence intervals were calculated using univariable Poisson regression; rates per 100 person-years follow-up (PYFU) were reported. Spearman's correlation coefficients were calculated to assess the relationships between number of Ng and relative time since vaccination. A 2-sided probability value (*P* value) < .05 Differences were considered statistically significant at *P* < .05. Statistical analyses were performed using R statistical software, version 4.2.2 (R Foundation for Statistical Computing).

## RESULTS

A total of 473 GBMSM vaccinated with 2 doses of 4CMenB were included in the study. The median follow-up (IQR) was 2.51 (1.36–3.27) years before and 4.39 (3.72–5.44) years after 4CMenB vaccination. The median age (IQR) at baseline was 43 (37–51) years. Eighteen of 473 (3.8%) were PrEP users, and 451 of 473 (95.3%) were people with HIV. Regarding HIV-related characteristics, all people with HIV has been receiving antiretroviral treatment for a median (IQR) of 7.22 (3.86–13.2) years, 46 of 451 (10.2%) had a previous AIDS event, and the median CD4^+^ lymphocyte nadir (IQR) was 346/µL (228–492/µL). HIV RNA levels were <50 copies/mL in 439 of 451 (97.3%) people with HIV, the median CD4^+^ lymphocyte count (IQR) was 792/µL (614–993/µL), and the median CD4^+^/CD8^+^ ratio was 0.89 (0.66–1.15) at baseline. Previous STIs were common in this cohort: 65 of 473 (13.7%) had a history of chlamydia infection (85 episodes, 74 at the rectal site), 118 of 473 (24.9%) has a history of syphilis, and 3 of 473 (0.6%) had a history of mpox. In total, 22 of 473 people (4.6%) had a history of active hepatitis B virus infection, and 3 (0.6%) had a history of hepatitis C virus.

The percentage of NAAT-positive rectal Ng swab samples was 76 of 957 (7.7%) 1 month after the second dose of 4CMenB vaccine and 51 of 456 (11.1%) before baseline (1413 total Ng swab samples in the study period). Breakthrough rectal Ng infections after baseline were 76 in 57/473 people. After 4CMenB the IR per 100 PYFU was 3.72 (2040 PYFU), and before 4CMenB the IR was 4.33 per 100 PYFU (1175 PYFU). Multiple episodes of breakthrough rectal Ng infections were observed in 13 of 57 people (22.8%): 9 of 57 (15.8%) had 2 episodes and 4 of 57 (7.0%) had 4. Episodes of NAAT-confirmed breakthrough rectal Ng also showed a positive Ng culture in 34 of 76 (44.7%), and 28 of 76 (36.8%) were symptomatic. Concurrent STIs were common: 22 of 76 (28.9%) had Ct (20 at rectal site); 5 of 76 (6.5%), Mgen (3 at rectal site); 4 of 76 (5.3%), syphilis; 4 of 76 (5.3%), mpox; and 1 of 76 (1.3%), genital HSV-1/2. Regarding HIV-related characteristics, at the time of breakthrough rectal Ng, in 58 of 64 cases (90.6%). HIV-RNA levels were <50 copies/mL, the median CD4^+^ lymphocyte count (IQR) was 808/µL (645–964/µL), and the median CD4^+^/CD8^+^ ratio was 0.98 (0.82–1.27).

Comparison of the characteristics of people with or without evidence of breakthrough rectal Ng after 4CMenB vaccination is shown in Table 1. People with rectal Ng after 4CMenB were significantly younger (medium age [IQR] with vs without breakthrough Ng, 38.2 [33.3–44.6] vs 44.2 [38.0–51.3] years; *P* < .001), more likely to be PrEP users (6 of 57 [10.5%] for breakthrough Ng vs 12 of 416 [2.9%] for no breakthrough Ng; *P* = .01), and less likely to live with HIV (51 of 57 [89.5%] vs 400 of 416 [96.2%], respectively; *P* = .04). Rectal Ng breakthrough infections were observed more frequently in people with a previous STI (41 of 57 [71.9%] for breakthrough Ng vs 184 of 416 [44.2%] for no breakthrough Ng; *P* < .001) and who had a higher number of sexual partners in the 3 months before baseline (>10 partners in 30 of 57 [53.4%] for breakthrough Ng vs 80 of 416 [19.2%] for no breakthrough Ng; *P* < .001]. Specifically, people with a history of chlamydia, gonorrhoea, and mycoplasma were more likely to have a breakthrough rectal Ng (Table 1).

**Table 1.** Baseline Characteristics of Individuals Vaccinated With 4CMenB According to the Presence or Absence of Breakthrough Rectal Gonorrhoea After Vaccination

Characteristic	Patients, No. (%)^a^			*P* Value
	Overall (n = 473)	Breakthrough Ng (n = 57)	No Breakthrough Ng (n = 416)	
Age^b^	43.4 (37.4–50.9)	38.2 (33.3–44.6)	44.2 (38.0–51.3)	**<.001^c^**
PrEP use^b^	18 (3.81)	6 (10.5)	12 (2.88)	**.01^c^**
Living with HIV^b^	451 (95.3)	51 (89.5)	400 (96.2)	**.04^c^**
Previous AIDS^b^	46 (9.73)	5 (8.77)	41 (9.86)	.98
HIV-RNA > 50 copies/mL^b^	15 (3.30)	2 (4.00)	13 (3.22)	.68
CD4^+^ cell count, median (IQR), cells/µL^b^	792 (614–993)	799 (608–996)	790 (616–990)	.72
CD4^+^/CD8^+^ ratio, median (IQR)^b^	0.89 (0.66–1.15)	0.94 (0.65–1.16)	0.89 (0.66–1.15)	.50
HCV antibody positive^b^	65 (13.7)	9 (15.8)	56 (13.5)	.06
HbsAg positive^b^	22 (4.65)	3 (5.26)	19 (4.57)	.81
Previous STI^d^	225 (47.4)	41 (71.9)	184 (44.2)	**<.001^c^**
Previous chlamydia^d^	65 (13.7)	15 (26.3)	50 (12.0)	**.006^c^**
Previous syphilis^d^	118 (24.9)	19 (33.3)	99 (23.8)	.16
Previous gonorrhoea^d^	59 (12.5)	15 (26.3)	44 (10.6)	**.002^c^**
Previous Mgen^d^	77 (16.3)	22 (38.6)	55 (13.2)	**<.001^c^**
Previous mpox^d^	3 (0.63)	0 (0.00)	3 (0.72)	>.99
Previous HSV^d^	33 (6.98)	7 (12.3)	26 (6.25)	.10
>10 Sexual partners^e^	110 (23.2)	30 (53.4)	80 (19.2)	**<.001^c^**

Abbreviations: HbsAg, hepatitis B surface antigen; HIV, human immunodeficiency virus; HSV, herpes simplex virus; IQR, interquartile range; Mgen, *Mycoplasma genitalium*; Ng, *Neisseria gonorrhoeae*; PrEP, preexposure prophylaxis; STI, sexually transmitted infection.

^a^Data represent no. (%) unless otherwise specified.

^b^At baseline.

^c^Significant at *P* < .05 (bold).

^d^In previous medical history.

^e^In the 3 months before baseline.

The characteristics of breakthrough rectal Ng infections compared with prevaccination rectal Ng episodes are shown in Table 2. Rectal Ng infections after 4CMenB vaccination were less likely to be symptomatic (26 of 76 [34.2%] after vs 34 of 51 [66.7%] before 4CMenB; *P* = .001) and more likely to have a negative gonococcal-specific culture (34 of 76 [44.7%] vs 41 of 51 [80.4%]; *P* < .001). There was no difference in the proportion of STIs concomitant to rectal Ng infection after versus before 4CMenB vaccination (28 of 76 [36.8%] vs 19 of 51 (37.3%), respectively; *P* > .99). In detail, no difference was observed with regard to concomitant syphilis (primary, secondary, or early latent), rectal mpox, rectal HSV-1/2, rectal Ct, or rectal Mgen (Table 2). When comparing the characteristics of rectal Ng infections after 4CMenB between people with or without rectal Ct coinfection, no difference was found in terms of presence of symptoms (12 of 20 [60%] with vs 30 of 56 [53.6%] without Ct coinfection; *P* = .8) or culture-positive Ng infections (13 of 20 [65%] vs 35 of 56 [62.5%], respectively; *P* > 99].

**Table 2.** Clinical and Microbiological Characteristics of Rectal Gonorrhoea Before and After 4CMenB Vaccination

Characteristic	Patients, No. (%)			*P* Value
	Overall (n = 127)	Ng Before 4CMenB (n = 51)	Ng After 4CMenB (n = 76)	
Positive Ng culture	75 (59.1)	41 (80.4)	34 (44.7)	**<.001^a^**
Presence of Ng symptoms	60 (47.2)	34 (66.7)	26 (34.2)	**.001^a^**
Concurrent STI (any)	47 (37.0)	19 (37.3)	28 (36.8)	>.99
Concurrent rectal chlamydia	32 (25.2)	12 (23.5)	20 (26.3)	.88
Concurrent rectal Mgen	7 (5.51)	4 (7.84)	3 (3.95)	.44
Concurrent rectal HSV	1 (0.79)	0 (0.00)	1 (1.32)	>.99
Concurrent rectal mpox	5 (3.94)	1 (1.96)	4 (5.26)	.65
Concurrent syphilis	10 (7.87)	6 (11.8)	4 (5.26)	.20

Abbreviations: HSV, herpes simplex virus; Mgen, *Mycoplasma genitalium*; Ng, *Neisseria gonorrhoeae*; STI, sexually transmitted infection.

^a^Significant at *P* < .05 (bold).

**Figure 1.**
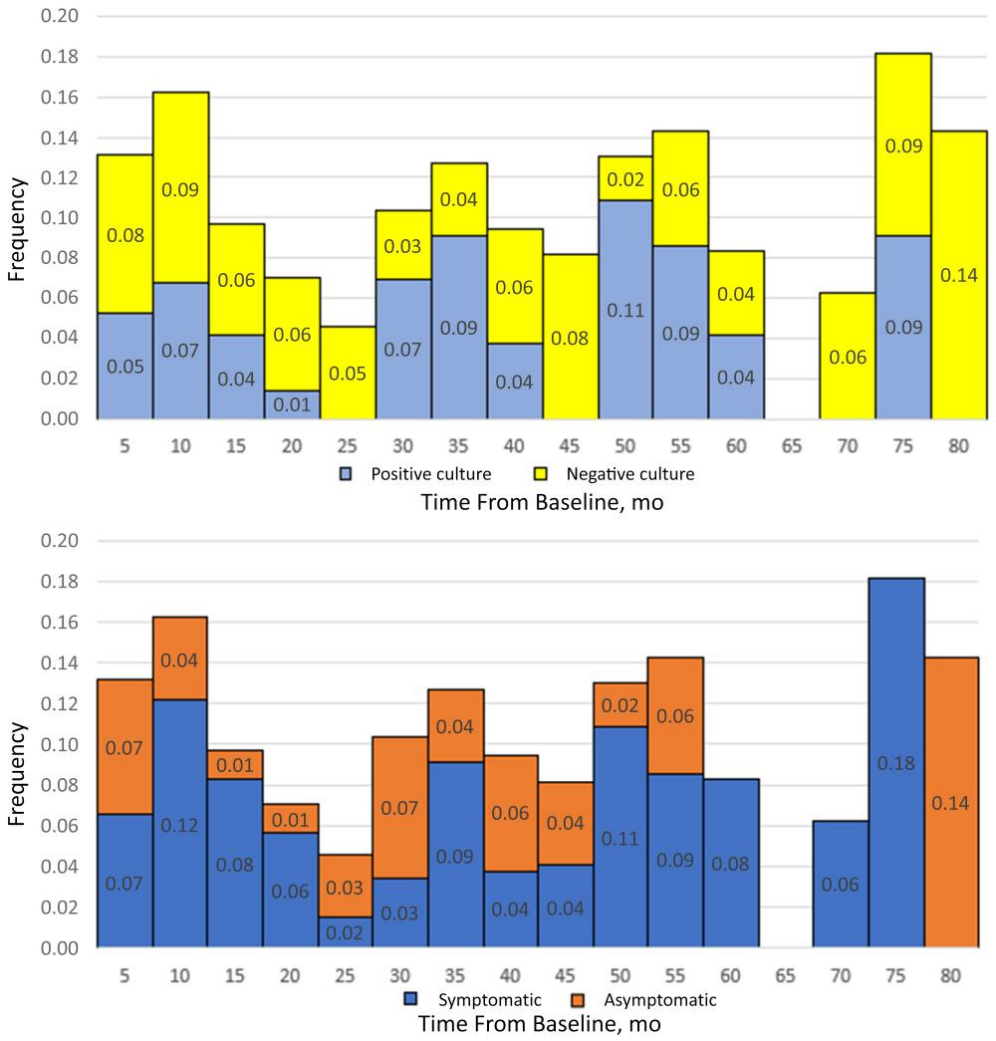
Timing of breakthrough rectal gonorrhoea infections (over the number of individuals at risk in each time interval) from 4CMenB vaccination (baseline), according to the presence or absence of symptoms and rectal gonorrhoea culture results.

The timeline of the events of breakthrough rectal Ng, over number of individuals at risk, from baseline is presented in Figure 1. We did not observe a significant association between the number of months after 4CMenB vaccination and the occurrence of breakthrough rectal Ng infection (overall, r = 0.007 and *P* = .98), even after adjusting for the presence of symptoms (symptomatic, r = −0.26 and *P* = .31; asymptomatic: r = −0.12 and *P* = .66) and evidence of positive Ng culture (positive: r = −0.15 and *P* = .57; negative, r = 0.08 and *P* = .77).

The use of doxycycline postexposure prophylaxis (DoxyPEP) was introduced at the Infectious Diseases Unit of the IRCCS San Raffaele Scientific Institute, Milan, at the end of 2022, based on available evidence and according to guidelines for prescribing on a case-by-case basis [18]. In total, 14 study participants were prescribed and subsequently used DoxyPEP at least once during study follow-up. In detail, 9 episodes of rectal gonorrhoea were observed among those who used DoxyPEP and were vaccinated with 4CMenB (3 episodes before vaccination and 6 after).

## DISCUSSION

In this study, breakthrough microbiologically confirmed rectal Ng infections were frequently identified in GBMSM following full 4CMenB vaccination, consistent with previous retrospective studies [5–9]. A higher incidence in this specific anatomic site may have been observed when compared with previous estimates of vaccine efficacy [5–9].

Most of the GBMSM enrolled in this study were people with HIV, as the 2017 version of the Italian HIV management guidelines already recommended vaccination with 4CMenB for all members of this core group due to the increased risk of invasive meningococcal disease. Most people with HIV had high levels of CD4^+^ lymphocytes and undetectable HIV-RNA at the time of meningococcal vaccination, and all were receiving antiretroviral treatment. Given this, reduced immunogenicity of the vaccine as a result of immunodeficiency was not expected, as has been shown in previous studies of protection against *N meningitidis* [19]. Fewer PrEP users received 4CMenB as a result of off-label vaccination after counseling. To properly address existing unresolved questions about the use of 4CMenB against Ng, we included only people who were screened for Ng to better address the key issue of vaccine effectiveness in relation to the presence of symptoms, and only people who reported having sex without a barrier method and were therefore at risk of rectal infection. In addition, only NAAT-confirmed cases of Ng were included to avoid misdiagnosis in the case of syndromic management.

GBMSM with breakthrough rectal Ng infections were indeed more likely to engage in higher-risk sexual behavior. A higher number of sexual partners and an increased number of previous STIs, including syphilis, chlamydia, and mpox, were observed in people who did not prevent rectal infection with 4CMenB. Not surprisingly, the vaccine was more likely to fail to protect against Ng in case of repeated and increased risk of exposure to Ng. 4CMenB vaccination shows sterilizing activity when its primary indication of invasive meningococcal disease is considered; however, given the hypothesized cross-protective immunity against Ng and previous evidence of partial efficacy, protection is likely to be limited and prone to failure in the case of substantial exposure to Ng. Indeed, we can highlight that breakthrough infections were clustered among members of this key population with the highest risk of STIs. In addition, we could hypothesize that in individuals at substantial risk of STIs with previous or active Ng infection, 4CMenB administration could lead to anergia or exhaustion, thus reducing the estimated efficacy within this core group. In fact, more people with breakthrough Ng infection had a previous Ng infection, although this could be due only to a generally higher risk profile for acquiring STIs.

Although we identified frequent breakthrough rectal Ng infections, these were less often symptomatic and more often with negative gonococcal-specific cultures, suggesting a possible lower virulence of rectal Ng after 4CMenB. Indeed, these results highlight that even if Ng is acquired, it may cause fewer adverse events due to the presence of fewer symptoms and may also have a role in infection control, as the negative cultures may represent a lower bacterial load and therefore lower infectivity. However, we emphasize that these data may only apply to the rectal site and may not represent the effect of 4CMenB on the urethral and pharyngeal sites. As suggested in another retrospective study [8], we did not observe an effect of rectal chlamydia coinfection on the proportion of symptomatic and culture-positive breakthrough rectal Ng infections.

When evaluating the timeline of breakthrough rectal Ng, we did not identify any clustering of infections or their characteristics (ie, symptoms and cultures) in relation to vaccination. This may be related to the long follow-up observed (up to 6.5 years), but as previously suggested in the New Zealand study, waning efficacy may take longer, and we do not currently have long follow-up data, as 4CMenB was licensed as an antimeningococcal vaccine relatively recently [10].

To date, the French ANRS DOXYVAC study (NCT04597424) is the only published clinical trial which investigated 4CMenB vaccination effectiveness on gonorrhoea infection [20]. The study, which was stopped early due to the efficacy of both DoxyPEP and 4CMenB vaccination at the interim analysis, showed in the final analysis that 4CMenB vaccination had a marginal effect on Ng, without statistical significance (adjusted hazard ratio for first Ng episode, 0.78 [95% confidence interval, .60–1.01]; adjusted IR ratio for cumulative Ng episodes, 0.84 [.67–1.07]) [20]. No vaccination effect was also observed in terms of culture-positive and symptomatic Ng infections, although these data were analyzed together at the rectal, urethral, and pharyngeal sites, with most infections being detection of asymptomatic pharyngeal Ng [20]. This may explain the discrepancy in the results of this study, which focused only on rectal Ng. We note that although previous observational studies were retrospective, a 2023 study assessed potential healthy vaccine bias and showed that the MenB-FHbp vaccine had no effect on gonorrhoea infections [21]. Similar to the findings in the DOXYVAC trial, which included only PrEP users, breakthrough Ng infections were more common in PrEP users than in people with HIV in this study [20].

In addition, as recently highlighted by White et al [22], the idea that 4CMenB is not valuable against Ng based on the results of the DOXYVAC trial alone can be challenged. As the trial was discontinued early, the study may have been underpowered to show moderate vaccine efficacy, which modelings suggest could still have a profound impact on Ng in certain key populations [20, 22]. We believe that, given the unique scenario of multiple clinical trials already underway evaluating this issue in different populations, with different end points and study designs (NCT04398849, NCT05766904, NCT05294588, NCT04722003, NCT04415424, NCT04350138, NCT05766904, NCT04597424, and NCT05766904), more evidence is needed to properly address open questions about whether this vaccination strategy should be implemented at the national level, especially given the limited information available on women.

Several limitations of this study must be acknowledged. First, this is a retrospective observational study, which is prone to bias. However, we believe that the rigorous clinical follow-up for HIV and PrEP treatment, together with the high frequency of STI testing during follow-up, validates our study findings. In addition, some participants were using DoxyPEP, which may have affected the detection of Ng. However, we note that very few people with low antimicrobial consumption used this preventive strategy, with a low risk of unsupervised drug use, given the favorable opinion on DoxyPEP implementation of our center and the recording of DoxyPEP use at each routine clinical visit. In addition, Ng tetracycline resistance can reach proportions of >90% in the European setting, rendering DoxyPEP ineffective against this specific STI [23]. Of note, the effect of DoxyPEP on rectal chlamydia infections may still have influenced the detection of rectal Ng, but, given the limited use of the prophylaxis, we believe the overall study results to be true.

There are also other potential confounding factors, such as the severe acute respiratory syndrome coronavirus 2 (SARS-CoV-2) pandemic occurring during the study period, resulting in changes in sexual behaviors and risk of STI acquisition. However, people were vaccinated with 4CMenB at various points during the study period between 2017 and 2023, which is likely to mitigate this confounding factor. In fact, the overall results of this study are consistent with previous observational reports. However, given the results of the DOXYVAC clinical trial, we believe that the various ongoing clinical trials will ultimately clarify the potential use of 4CMenB against Ng.

In conclusion, this study found overall that 4CMenB could play a role in controlling Ng rectal infections. Breakthrough rectal Ng infections following 4CMenB vaccination were preferentially identified in people with higher-risk sexual behaviors and were less often symptomatic and more often with negative gonococcal-specific cultures, suggesting a possible lower virulence of the infection.
