# Correction to: Breakthrough Rectal *Neisseria gonorrhoeae* Infections After Meningococcal B Vaccination: Microbiological and Clinical Features

**DOI:** 10.1093/ofid/ofaf350

**Published:** 2025-06-19

This is a correction to: Angelo Roberto Raccagni, Sara Diotallevi, Riccardo Lolatto, Elena Bruzzesi, Maria del Carmen Garcia Martearena, Ilaria Mainardi, Caterina Candela, Diana Canetti, Girolamo Piromalli, Nicola Clementi, Roberto Burioni, Antonella Castagna, Silvia Nozza, Breakthrough Rectal *Neisseria gonorrhoeae* Infections After Meningococcal B Vaccination: Microbiological and Clinical Features, *Open Forum Infectious Diseases*, Volume 11, Issue 11, November 2024, https://doi.org/10.1093/ofid/ofae562

In the originally published version of the manuscript, two names of the fifth co-author were erroneously transposed. This name is now emended in the article to read: “Maria del Carmen Garcia Martearena”.

